# Central Nervous System Fungal Diseases in Children with Malignancies: A 16-Year Study from the Infection Working Group of the Hellenic Society of Pediatric Hematology Oncology

**DOI:** 10.3390/jof10090654

**Published:** 2024-09-14

**Authors:** Loizos Petrikkos, Maria Kourti, Kondylia Antoniadi, Tatiana-Sultana Tziola, Angeliki-Eleni Sfetsiori, Vasiliki Antari, Sofia Savoukidou, Georgia Avgerinou, Maria Filippidou, Eugenia Papakonstantinou, Sophia Polychronopoulou, Emmanuel Hatzipantelis, Dimitrios Doganis, Antonios Kattamis, Vassilios Papadakis, Emmanuel Roilides, Athanasios Tragiannidis

**Affiliations:** 1 Department of Pediatric Hematology-Oncology (T.A.O.), “Aghia Sophia” Children’s Hospital, 11527 Athens, Greece; condilia.and@gmail.com (K.A.); sophpol@otenet.gr (S.P.); vpapadak@otenet.gr (V.P.); 2 Infectious Diseases Unit, 3rd Department of Pediatrics, Hippokration General Hospital, School of Medicine, Aristotle University of Thessaloniki, 54642 Thessaloniki, Greece; roilides@auth.gr; 3 Pediatric Hematology-Oncology Unit, 1st Pediatric Department, “Aghia Sophia” Children’s Hospital, National and Kapodistrian University of Athens, 11527 Athens, Greece; t.s.tziola@hotmail.com (T.-S.T.); g.avgerinou@yahoo.gr (G.A.); m.filippidou@hotmail.com (M.F.); ankatt@med.uoa.gr (A.K.); 4 Oncology Department, “P & A Kyriakou” Children’s Hospital, 11527 Athens, Greece; angelina.sfe@gmail.com (A.-E.S.); doganisd@gmail.com (D.D.); 5 Children & Adolescent Hematology-Oncology Unit, Second Department of Pediatrics, Hippokration General Hospital, School of Medicine, Aristotle University of Thessaloniki, 54124 Thessaloniki, Greece; antarivaso@gmail.com (V.A.); hatzip@auth.gr (E.H.); atragian@auth.gr (A.T.); 6 Department of Pediatric Oncology, Hippokration General Hospital, 54642 Thessaloniki, Greece; sof_savvouk@hotmail.com (S.S.); eugepapa@yahoo.gr (E.P.)

**Keywords:** central nervous system, invasive fungal infection, children, malignancies, outcome, brain abscess

## Abstract

We analyzed data on pediatric invasive fungal diseases of the central nervous system (CNS-IFDs) reported by five of a total of eight Pediatric Hematology-Oncology Departments in Greece for 16 years (2007–2022). A total of twelve patients (11 boys, median age: 9.5 years, range: 2–16) were reported suffering from CNS-IFDs. The underlying malignancy was acute lymphoblastic leukemia in 9/12 and acute myeloid leukemia, Ewing sarcoma, and rhabdomyosarcoma in one each. Eleven patients presented with CNS-related symptoms (i.e., seizures, headache, cerebral palsy, ataxia, hallucination, seizures, blurred vision, amaurosis). All patients had pathological MRI findings. Multifocal fungal disease was observed in 6/12 patients. Nine proven and three probable CNS-IFD cases were diagnosed. Causative pathogens in proven cases were *Aspergillus* spp. and *Candida albicans* (n = 2 each), *Mucor* spp., *Rhizopus arrhizus*, *Absidia* spp., *Fusarium oxysporum* and *Cryptococcus neoformans* (n = 1 each). Causative pathogens in probable cases were *Aspergillus* spp. (n = 2) and *Candida* spp. (n = 1). All patients received appropriate antifungal therapy (median duration: 69.5 days, range 19–364). Two patients underwent additional surgical treatment. Six patients were admitted to the Intensive Care Unit due to complications. Three patients (25%) died, two due to IFD and one due to an underlying disease. Early recognition and prompt intervention of CNS-IFDs may rescue the patients and improve overall survival.

## 1. Introduction

Invasive fungal diseases (IFDs) of the central nervous system (CNS) pose significant clinical challenges, especially in immunocompromised populations. The epidemiology of these infections is shifting, with a marked increase in both the incidence and the diversity of the causative agents. A study on pediatric patients with CNS tumors reported a 4.6% incidence of IFDs, predominantly caused by *Candida* species (90.9%) and *Aspergillus* spp., particularly in children undergoing chemotherapy or neurosurgery [1]. Filamentous fungi, mostly *Aspergillus* spp. but also *Fusarium* spp. and Mucorales may affect the CNS in children [2,3,4,5,6,7]. Clinical symptoms are often unusual or atypical and may even be absent in a significant proportion of patients with IFDs of the CNS (CNS-IFDs), which makes early diagnosis and prompt initiation of antifungal treatment difficult. Among the neurological symptoms, hemi- or quadriplegia, seizures, headache, cerebral palsy, ataxia, hallucination, seizures, and blurred vision or amaurosis have been described [3,4,7,8]. Diagnosing CNS-IFDs is particularly challenging due to nonspecific symptoms and the limitations of imaging techniques. While MRI is the preferred imaging modality, it often lacks definitive findings [6]. Laboratory diagnostics include cultures and serological assays, like 1,3-beta-D-glucan in cerebrospinal fluid, that have an emerging role in detecting and monitoring CNS-IFDs [9]. Effective management requires prompt antifungal therapy, with liposomal amphotericin B and voriconazole being the common choices [6]. The treatment duration is guided by clinical and radiological responses [6].

The emergence of antifungal resistance is particularly concerning with *Candida* species, where strains exhibit varying susceptibility to commonly used antifungals such as fluconazole and voriconazole [10]. This resistance, coupled with the limited efficacy of current antifungal therapies and their poor CNS penetration, significantly complicates treatment strategies. Since the landscape of antifungal resistance in CNS infections is increasingly complex, driven by the rise of immunocompromised populations and the limitations of current antifungal therapies, there is an urgent need for novel antifungal agents and combination therapies to overcome the limitations of current treatments and effectively combat antifungal resistance.

This study aims to present the data of pediatric CNS-IFD cases from eight Greek pediatric Hematology-Oncology Departments during a 16-year period (2007–2022) that were collected and analyzed by the Infection Working Group (IWG) of the Hellenic Society of Pediatric Hematology-Oncology.

## 2. Patients and Methods

The study was retrospectively conducted in the eight pediatric Hematology-Oncology Departments and Units in Greece. The units are the Department of Pediatric Hematology-Oncology and the Pediatric Hematology-Oncology Unit of the First Department of Pediatrics of the “Aghia Sophia” Children’s Hospital in Athens, the Oncology Department of the “P. & A. Kyriakou” Children’s Hospital in Athens, the Pediatric and Adolescent Oncology Clinic of “MITERA” Hospital in Athens, the Children and Adolescent Hematology-Oncology Unit of the Second Department of Pediatrics in the AHEPA Hospital in Thessaloniki, the Department of Pediatric Oncology of the “Hippokration” Hospital in Thessaloniki, the Pediatric Hematology Oncology Department of the General University Hospital of Heraklion in Crete, and the Stem Cell Transplant Unit of the “Aghia Sophia” Children’s Hospital in Athens. In Greece, nearly 300 pediatric oncology patients between 0 and 14 years old are diagnosed each year, within a national population of approximately 11 million. The study consisted of the collection of clinical and laboratory data, clinical courses, and outcomes of the CNS-IFD cases diagnosed in pediatric hematology-oncology patients (0–16 years) for the period 2007–2022.

Pediatric patients receiving chemotherapy and/or radiotherapy for an underlying malignancy were included in the study. The study eligibility criteria of proven or probable fungal infection according to the European Organization for Research and Treatment of Cancer/Mycosis Study Group (EORTC/MSG) were used [11]. According to these criteria, proven CNS fungal infection was diagnosed by CNS imaging or a macroscopic autopsy finding in conjunction with a positive microbiological result in the brain tissue or the cerebrospinal fluid (CSF). Positive microbiological results included a positive culture, microscopic evidence of hyphae, a positive galactomannan (GM) test, or a positive polymerase chain reaction (PCR) for a fungus. Probable fungal infection was defined when compatible CNS imaging findings were combined with proven or probable invasive fungal infections at a site outside the CNS. 

The sites of fungal infections were classified as localized when confined in CNS only (isolated or disseminated in CNS) or multifocal when the infection was disseminated to two or more non-contiguous locations/organs.

The outcome was defined as survival or death at the last contact of follow-up. Mortality was assessed as all-cause mortality during the course of the fungal infection.

The medical records of the patients were thoroughly reviewed retrospectively in order to extrapolate and report all appropriate information and data, including demographic characteristics, type and stage of underlying malignant disease, potential risk factors (prolonged neutropenia, prior treatment with corticosteroids, type of chemotherapy, other treatment modalities, hospitalization for more than 20 days, prior ICU stay, comorbidities), clinical symptoms, site(s) of infection, radiological findings (brain and spine MRI), laboratory findings, treatment (antifungal drug therapy, previous prophylactic antifungal therapy, surgery, duration of the treatment), outcome, and subsequent sequelae.

## 3. Results

During the study period, twelve cases of CNS-IFDs were recorded and reported from five out of the eight Pediatric Hematology-Oncology Departments existing in Greece. There were eleven males and one female with a median age of 9.5 years at the time of infection (range: 2–16 years).

Acute lymphoblastic leukemia (ALL) was the primary underlying malignancy in nine patients, whereas acute myeloid leukemia (AML), Ewing sarcoma, and rhabdomyosarcoma in one patient each. Among the nine patients with ALL, one child suffered from relapsed ALL post hematopoietic stem cell transplantation (HSCT) and one from refractory ALL.

All patients received intensive chemotherapy for their underlying malignancy according to current international protocols: six patients were treated according to the ALLIC BFM 2009, two according to the AIEOP-BFM ALL 2017, one based on the ALLIC REL Guidance 2016, one based on the AML BFM 2004, one with iEuroEwing and one with the RMS88 Protocol (Table 1).

Seven out of twelve patients (58.3%) had been hospitalized for more than 20 days. Seven patients with ALL (58.3%) had received prolonged corticosteroid treatment, and all presented with severe neutropenia with a neutrophil count <500 μL at the time of diagnosis.

Eleven (11/12) patients presented with one or more CNS-related symptoms: ten patients presented with headache (in 4/10 as the only CNS-related presenting symptom), four with seizures (in 1/4 as the only CNS-related presenting symptom), two each with cerebral palsy or blurred/impaired vision, one each with ataxia or with hallucinations, and six with various other symptoms such as hypotonia, coma or irritability.

**Figure 1:**
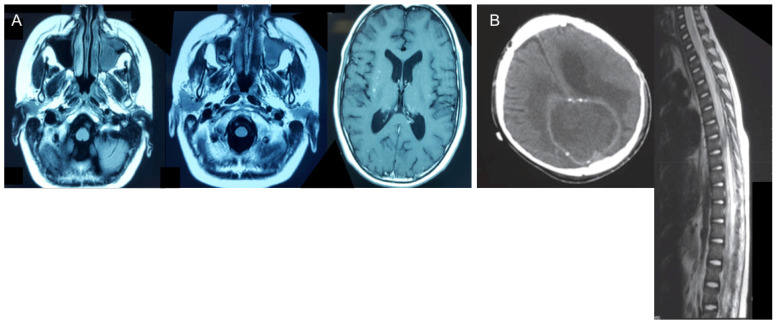
Abnormal MRI findings. (**A**) Scattered speckled foci in the brain, sinuses, and left orbit in Patient 4. (**B**) Subdense brain lesion plus bleeding and lesions in the spinal cord of Patient 2.

Abnormal MRI findings were found in all patients: brain abscess in four patients; meningoencephalitis/meningitis in three patients; granuloma in one patient; sub-dense brain lesions in four patients; edema of ethmoid cells, frontal sinuses, and frontal lobe infiltration in one patient; and soft tissue in the visceral skull region and part of the frontal sinus in one patient (Figure 1).

Multifocal fungal infection was observed in six patients (50%). Concomitant lung involvement was revealed in three cases, sinus involvement in four, and liver involvement in one case. Disseminated disease was diagnosed in eight out of twelve patients (66.7%). Neurological symptoms, MRI findings, and evidence of disease (proven, probable) according to diagnostic tools are shown in Table 2.

Nine proven CNS-IFDs and three probable cases were diagnosed (Table 3). Proven IFD was documented by histology or culture in all nine patients. Causative pathogens in proven cases included three *Mucorales*, two *Aspergillus* spp., two *Candida albicans*, one *Fusarium oxysporum,* and one *Cryptococcus neoformans*. Causative pathogens in probable cases included two *Aspergillus spp.*, with positive serum galactomannan test in both cases, and one *Candida spp.* with elevated serum 1,3-beta-D-glucan levels.

Nine patients (75%) had received antifungal prophylaxis before the diagnosis of CNS IFD. The majority of patients underwent antifungal prophylaxis with fluconazole or micafungin (8/9 patients). All patients received appropriate therapy according to the isolated pathogen; the mean duration of therapy was 130 days (median: 69.5 days, range: 19–364 days). Antifungal therapy included liposomal amphotericin B (LAmB) at doses varying between 3–10 mg/kg/day in all patients (12/12) alone or combined with other antifungal agents. Liposomal amphotericin B alone was given in three out of twelve patients (two with *Aspergillus* spp. and one with *Cryptococcus neoformans*). Combined antifungal therapy was administered to nine patients, and the most common combinations were LAmB plus voriconazole (six patients) and LAmB plus posaconazole (two patients). Two patients underwent additional surgical treatment. Six cases were complicated and required ICU admission. Three of the twelve patients (25%) died, two patients due to IFD (16.7%) and one due to underlying disease.

## 4. Discussion

Invasive mycoses of the CNS are severe forms of infections associated with significant morbidity and mortality [3,4,5,6,7]. The frequency of IFDs has seen a notable increase over the last decades, but there is still a scarcity of epidemiological data concerning CNS-IFDs in pediatric populations [3]. Although there are newer diagnostic tools and antifungal agents for treatment, CNS-IFDs are still difficult to diagnose and treat promptly and successfully. In a multicenter retrospective study of pediatric patients with cancer, Cesaro et al. reported an incidence of 3.3% of CNS-IFDs [12]. In another prospective surveillance study focusing on adults, 14.1% of patients with hematological malignancies were diagnosed with invasive mold infections (IMD) affecting the CNS [5].

Numerous risk factors for IFDs among pediatric patients with hemato-oncological diseases have been identified in a recent systematic review by Fisher et al. [13]. These include underlying diseases such as AML, high-risk ALL or relapsed leukemia, prolonged and high-dose corticosteroid use, prolonged and profound neutropenia, comorbidities, and advanced age. In our patient cohort, out of the twelve individuals diagnosed with CNS-IFDs, ten had acute leukemia, with nine having ALL and one having AML. Among the ALL cases, two patients suffered from relapsed or refractory disease. While patients with ALL generally have a lower risk of IFDs compared to those with AML or leukemia relapse, ALL still represents the largest group of patients at risk for IFDs [14]. Moreover, in our patient cohort, all cases of CNS-IFDs occurred during intensive chemotherapy phases of treatment, characterized by profound neutropenia (mean absolute neutrophil count <500/μL). Prolonged corticosteroid treatment was also observed in seven out of twelve patients.

*Aspergillus* species were found to be the most frequently identified fungi in our cohort (two proven and two probable cases), with other rare molds, especially Mucorales, following in frequency (all proven cases). In general, molds were by far the most frequent pathogens causing CNS-IFDs compared to yeasts (eight vs. four cases). Invasive mold infections mainly affect the lungs but may also occur in other sites, such as the liver, kidney, bones, or the CNS. Involvement of the CNS represents an especially severe form of fungal infection, posing challenges not only in diagnosis but also in treatment. The significant occurrence of CNS involvement in pulmonary mold infections has led to the recommendation in the most recent European Conference on Infections in Leukemia (ECIL) guidelines to contemplate CNS imaging in patients with pulmonary mold infection, even in the absence of neurological symptoms [11]. In our study, lung involvement was revealed in three cases, liver involvement in one, and sinus involvement in four. This observation corroborates the etiopathogenic hypothesis that IFDs affecting the CNS could stem from the hematogenous dissemination of an infection originating from another primary site, primarily the lung [4,15]. In a report from the Israeli Study Group of Childhood Leukemia, 59% of patients with mucormycosis presented with a rhinocerebral pattern of infection, with eight of them showing adjacent spread to the CNS [16].

In our series of CNS-IFDs, eleven out of the twelve patients exhibited symptoms related to the CNS, with headache being the most prominent among them (83.3%, 10/12 patients), followed by seizures in 30%. In four patients, headache was the only presenting CNS-related symptom, while seizures were present in one patient. Nevertheless, symptoms may be non-specific and encompass a range of focal and non-focal neurological manifestations. Additionally, the percentage of symptomatic patients and the type of symptoms may vary in studies due to differences in registries. In our cohort, one patient had no CNS-related symptoms but abnormal brain MRI findings. Indeed, it is intriguing to note that almost one-third of children with CNS invasive mold infections were neurologically asymptomatic in a study involving 29 children, and CNS involvement was detected through diagnostic evaluations for pulmonary or liver fungal infections or incidentally during routine CNS imaging [8]. This highlights the importance of CNS imaging in patients with proven or probable mold infection outside the CNS. In a retrospective study of 19 pediatric patients with proven or probable CNS-IMD, fungal abscesses and parenchymal inflammation (cerebritis) were the most frequently observed features [7]. In our cohort of patients, abscesses were revealed in 4 patients, while meningoencephalitis/meningitis was found in three patients. The edema of ethmoid cells, frontal sinuses, and frontal lobe infiltration in one patient, and soft tissue in the visceral skull region and part of the frontal sinus in one patient, reveals the initial mucosal inflammation and angio-invasive nature of the fungus leading to direct extension through vascular invasion. Nevertheless, a definitive diagnosis of CNS-IFDs entails cultures obtained through sterile procedures and/or histopathological, cytopathological, or direct microscopic evaluations [4,17]. Proven IFD was documented by histology or culture in all nine patients of our cohort. Several non-culture-based assays have been developed as adjunctive diagnostic tools; the ECIL guidelines recommend GM evaluation in the CSF in immunocompromised patients with suspected CNS invasive mold disease, with a proposed threshold of 1.0 [18]. However, it is crucial to interpret the test results carefully, as there are limitations, such as poor positive predictive values. Since data on molecular assays are limited and heterogeneous in the pediatric setting, the current ECIL guidelines have not included molecular testing-specific recommendations for children [18].

Initiating effective antifungal treatment promptly remains essential for the therapeutic management of CNS-IFDs. Recent pediatric guidelines suggest voriconazole as the preferred treatment for invasive aspergillosis (A-It) and LAmB as an alternative in case of suspected or confirmed azole resistance (B-It) [18]. It is important to mention that voriconazole is not approved for children under two years old, and therapeutic drug monitoring is highly recommended to maintain serum levels >1–2 mg/L [17,18]. LAmB, on the other hand, is approved for children of all ages. The most used therapeutic agents in our series were LAmB, followed by voriconazole. Interestingly, all our patients received LAmB alone or combined with other antifungal treatments. The concurrent utilization of combined antifungal therapy lacks clinical endorsement or support from existing guidelines but is widely used when management reasoning eclipses evidence-based medicine [18]. Therefore, in numerous cases, including ours, multidrug treatment was administered due to clinicians’ concerns regarding systemic dissemination and the significant morbidity associated with IFDs, which may also lead to delays in chemotherapy. Despite the poor penetration through the blood-brain barrier due to their high molecular mass and protein binding, echinocandins were used in combination with LAmB in two patients with *Candida albicans* [19]. Despite micafungin not being detectable in the CSF, therapeutically effective levels of micafungin in brain tissue of rabbits could be achieved during tissue inflammation and/or necrosis, as demonstrated by the successful clearance of *C. albicans* from the CNS [20,21].

From a therapeutic perspective, our study reaffirms that surgical intervention has been employed in a minority of cases (two patients with mucormycosis and one with fusariosis). A multidisciplinary approach, including surgical intervention, is an important pillar for the successful management of disseminated mucormycosis and should be deliberated on a case-by-case basis [3,22]. In children with CNS mucormycosis, high-dose LAMB (5–10 mg/kg) is the treatment of choice. 

Twenty-five percent of the patients died in our cohort, including one patient who died from an underlying hematological disease. It is important to acknowledge that rates may vary across different studies, influenced by factors such as underlying disease, phase of anticancer treatment, or patient characteristics [2,23,24]. In addition to prompt initiation of effective antifungal treatment for IFDs, guidelines advocate for the management of predisposing conditions in both adults and children, such as severe neutropenia or immunosuppressive treatment [17,18].

We acknowledge various limitations in our study, such as the lack of standardized procedures in diagnostics and management strategies during the last 16 years at different centers. However, this variability highlights the need for optimization of antifungal therapy in children with hemato-oncological malignancies worldwide. Variations between centers in both the initiation and duration of antifungal therapy underscores an important research gap. However, it is worth emphasizing that our study offers valuable insights into the epidemiology and outcomes of CNS-IFDs in children, and it is the first study addressing this field. Our analysis possesses distinctive and significant strengths. We included only patients with proven and probable CNS-IFDs, and we provided information about the use of antifungal prophylaxis.

CNS-IFDs should be suspected even in the absence of neurological signs, as described in one patient of our cohort, and we suggest performing a routine diagnostic evaluation in suspected immunocompromised children. Our study presents the first nationwide cohort focused on CNS-IFDs. Although it is confirmatory of previous findings, we mentioned that mold cases were twice as common as yeast cases and that only two patients died due to CNS-IFDs.

Despite promising advancements in understanding and treating CNS IFDs in children, significant challenges persist in diagnosis and management, highlighting the need for continued research and development in this crucial area. Several novel antifungals are in the pipeline at various clinical stages, but translating clinical study results into practice remains challenging. In the interim, dedicated antifungal stewardship programs for immunocompromised children with hemato-oncological diseases are essential to optimize therapeutic outcomes, enhance patient care, and minimize the emergence of fungal resistance.

## Figures and Tables

**Table 1:** Demographic and clinical data, including predisposing factors.

Case	Sex	Age (yrs.)	Underlying Disease	Protocol	Disease Phase	Neutropenia	Hospitalization > 20 d	Chemo	Cortisone	Prophylaxis
1	M	16	Common B-ALL	ALL-IC BFM 2009	Induction	yes	no	yes	yes	yes
2	F	2	AML	AML 2004	Refractory	yes	yes	yes	no	yes
3	M	15	Relapsed ALL after HSCT	ALL-IC REL 2016	Induction	yes	yes	yes	yes	yes
4	M	11	Refractory ALL	ALL-IC BFM 2009	Refractory	yes	yes	yes	no	yes
5	M	8	ALL	ALL-IC BFM 2009	Induction	yes	yes	yes	yes	yes
6	M	11.5	T-ALL	AIEOP-BFM ALL 2017	Protocol ΙΒ1 + 2 long	yes	no	yes	yes	no
7	M	15	T-ALL	AIEOP-BFM ALL 2017	Protocol ΙΒ1 + 2 long	yes	no	yes	no	yes
8	M	6.75	Sarcoma Ewing	iEuroEwing	Protocol R2 IEVC-ΙΕ	yes	no	yes	no	no
9	M	5	B-ALL	ALL-IC BFM 2009	Protocol M	yes	no	yes	yes	no
10	M	2	B-ALL	ALL-IC BFM 2009	Protocol I	yes	yes	yes	yes	no
11	M	3.2	ALL	ALL-cIC BFM 2009	Induction	yes	yes	yes	yes	yes
12	M	11	RMS alveolar	RMS88 Protocol	Chemotherapy	yes	yes	yes	no	no

Abbreviations: HSCT (hematopoietic stem cell transplantation), RMS (rhabdomyosarcoma).

**Table 2:** MRI findings and evidence of disease (proven, probable) according to diagnostic tools.

Case	Neuro-Symptoms	MRI Findings	Multifocal IFD	Proven/Probable IFD	Biopsy/CSF	Culture	PCR	Biomarkers	Pathogens
1	yes	yes	yes	proven	yes	no	yes	-	*Aspergillus* spp.
2	yes	yes	no	proven	yes	yes	no	no	*Absidia* spp.
3	yes	yes	yes	probable	no	no	no	yes	*Candida* spp.
4	yes	yes	yes	probable	no	no	yes	yes	*Aspergillus* spp.
5	yes	yes	yes	proven	yes	yes	no	no	*Mucor* spp.
6	no	yes	no	proven	yes	-	-	-	*Candida albicans*
7	yes	yes	yes	proven	yes	yes	-	-	*Fusarium oxysporum*
8	yes	yes	no	proven	yes	yes	-	-	*Candida albicans*
9	yes	yes	no	proven	yes	no	yes	-	*Cryptococcus neoformans*
10	yes	yes	no	probable	no	no	no	yes	*Aspergillus* spp.
11	yes	yes	yes	proven	yes	no	-	-	*Rhizopus arrhizus*
12	yes	yes	no	proven	yes	-	-	-	*Aspergillus* spp.

**Table 3:** Pathogens isolated from the cases. A: proven; B: probable.

**A. Proven Pathogens**	**No**
*Aspergillus* spp.	2
*Mucor* spp.	1
*Rhizopus arrhizus*	1
*Absidia* spp.	1
*Fusarium oxysporum*	1
*Candida albicans*	2
*Cryptococcus neoformans*	1
**B. Probable Pathogens**	**No**
*Aspergillus* spp.	2
*Candida* spp.	1

## Data Availability

The data presented in this study are available on request from the corresponding author. The data are not publicly available due to privacy and ethical restrictions.
